# Forensic Age Estimation of the Knee with 3D MEDIC MRI

**DOI:** 10.3390/diagnostics16162641

**Published:** 2026-08-19

**Authors:** Fatma Celik Yabul, Elif Hocaoglu, Claire Villard, Eric Baccino, Sophie Colomb, Laurent Martrille

**Affiliations:** 1 Department of Radiology, Bakırkoy Dr. Sadi Konuk Training and Research Hospital, Health Sciences University, Istanbul 35000, Türkiye; fatmayabul@gmail.com (F.C.Y.); drelifhocaoglu@hotmail.com (E.H.); 2 Department of Legal Medicine, CHU Montpellier, University of Montpellier, 34000 Montpellier, France; claire.villard@chu-montpellier.fr (C.V.); ericbaccino@expertbaccino.fr (E.B.); s-colomb@chu-montpellier.fr (S.C.); 3 L’Équipe de Droit Pénal et de Sciences Forensiques de Montpellier (EDPFM), University of Montpellier, 34000 Montpellier, France; 4 ADES, UMR 7268, Aix-Marseille Université, Bloc A, 13344 Marseille, France

**Keywords:** forensic age estimation, knee MRI, 3D MEDIC, gradient-echo sequence, Dedouit classification, growth plate closure, diagnostic accuracy

## Abstract

**Background/Objectives:** Forensic age estimation increasingly relies on non-ionizing magnetic resonance imaging (MRI) evaluation of the knee growth plates. The original five-stage classification proposed by Dedouit et al. was developed using spin-echo proton-density-weighted imaging, and its applicability to gradient-echo sequences with different contrast mechanisms has not been fully established. This study aimed to apply the original, unmodified Dedouit classification to a volumetric three-dimensional Multiple Echo Data Image Combination (3D MEDIC) sequence and to generate corresponding age thresholds in a Turkish sample. **Methods:** Knee MRI examinations of 309 individuals (153 males, 156 females; age range 9.18–25.97 years) were retrospectively evaluated at a 3-Tesla field strength. Two experienced observers independently staged the distal femoral and proximal tibial epiphyses; intra- and inter-observer agreement were assessed using Cohen’s kappa. Spearman’s correlation and the Mann–Whitney U test, with rank-biserial effect sizes and Bonferroni correction, were used to assess the relationship between age and stage and between-sex differences, respectively. To provide forensically applicable thresholds, we additionally modeled the age at which the probability of complete fusion (Stage V) reached 50%, with 95% bootstrap confidence intervals, and calculated the sensitivity, specificity, and area under the curve (AUC) of Stage V for identifying individuals ≥18 years. **Results:** Agreement was very good for both epiphyses (kappa = 0.808–0.833). Age correlated strongly with stage for both the femur (rho = 0.70–0.76) and tibia (rho = 0.68–0.74). The 50%-probability age for complete fusion ranged from 15.23 years (tibia, females) to 17.43 years (femur, males). Stage V showed high sensitivity (0.96–0.98) but markedly lower and sex-dependent specificity for the 18-year threshold (0.48–0.58 in females versus 0.77–0.85 in males), indicating that Stage V alone is a poor sole criterion for confirming adult status in females. The youngest age at which Stage V was observed was 14.67 years in females and 16.07–16.16 years in males, markedly younger than thresholds reported using spin-echo imaging in the original Dedouit cohort. However, as an extreme-value statistic based on a single individual, this minimum should not be used as a forensic threshold in isolation. **Conclusions:** The Dedouit classification remains reproducible when applied to a 3D gradient-echo sequence, but the resulting age thresholds may be influenced by acquisition technique, population-specific factors such as socioeconomic status, or both. Consequently, thresholds derived from different MRI sequences or populations should not be assumed to be interchangeable without local validation.

## 1. Introduction

Forensic age estimation is required whenever an individual’s legal status—criminal responsibility, custody, or access to age-dependent protections in migration proceedings—cannot be established from documentary records [1,2]. Standard protocols combine radiographic assessment of the hand, dentition, and clavicle [3]. Because these rely on ionizing radiation and forensic age estimation is not a medical indication, their use in living individuals raises ethical and legal concerns [3,4], driving growing interest in MRI as a radiation-free alternative [4,5]. As in other forensic age-estimation modalities—including third-molar dental staging, where the concept of an ethically and technically acceptable margin of error has been explicitly discussed [6,7]—any MRI-derived threshold must be reported together with a quantified error rate rather than as a single cut-off age, since the practical consequence of a misclassification is a legal determination, not merely a measurement error.

MRI does not simply replace radiography; it assesses a different tissue. Radiographic and CT-based systems track cortical bone fusion, whereas MRI visualizes the cartilage of the growth plate directly, and the contrast mechanism used to do so differs by sequence. Spin-echo sequences—including the FSE PD protocol underlying Dedouit et al.’s original five-stage knee classification [8]—generate contrast from T2 relaxation with active compensation for field inhomogeneity. Gradient-echo (GRE) sequences instead rely on T2* relaxation, which is inherently more sensitive to susceptibility and chemical-shift effects. Multi-echo GRE protocols combine several gradient-recalled echoes into a single image and can be acquired in two-dimensional or volumetric three-dimensional form, the latter offering thinner contiguous sections at the cost of longer acquisition times.

These technical differences have measurable consequences. Comparisons of T1-, T2-, and proton-density-weighted sequences at the proximal humerus have shown systematically different stage transitions in the same population, prompting calls for sequence-specific reference thresholds [9]. Similar sequence-dependent discrepancies have been reported at the wrist [10] and, directly relevant here, at the knee itself, where cartilage-sensitive and T1-weighted sequences have yielded different minimum age estimates for growth-plate closure [11]. The Dedouit classification has already been re-applied at 3 Tesla [12] and, in our own prior work, using a two-dimensional multi-echo GRE sequence that required additional sub-stages to remain discriminative at older ages [13]. Sequence choice, in other words, is not a neutral methodological detail.

Population-specific data compound this picture: skeletal maturation timing varies by genetic and socioeconomic background [4], and Turkish knee MRI data remain limited to spin-echo and two-dimensional GRE protocols [14,15], leaving a volumetric multi-echo GRE acquisition untested in this population.

The present study applied the original, unmodified five-stage Dedouit classification to distal femoral and proximal tibial cartilage imaged with a volumetric multi-echo gradient-echo sequence in a Turkish sample, aiming to (i) establish forensically applicable age thresholds—expressed both as extreme-value minimum ages and, more robustly, as the age at which the probability of complete fusion reaches 50% together with sensitivity/specificity for the 18-year threshold—under this acquisition protocol, and (ii) compare the resulting thresholds descriptively against those reported using other sequences and populations in the literature. We did not perform a direct within-subject comparison of imaging sequences, so any inference about whether sequence choice per se, independent of population, shapes MRI-based age estimation of the knee remains a hypothesis for future prospective work rather than a conclusion supported directly by the present single-sequence design.

## 2. Materials and Methods

### 2.1. Sample Description

This study was carried out at Bakırköy Dr. Sadi Konuk Training and Research Hospital, Istanbul, Turkey. We retrospectively evaluated knee MRI examinations performed at the hospital between 2016 and 2019, together with the corresponding clinical records. All examinations had been requested for clinically indicated reasons—trauma, pain, or restricted range of motion—rather than for research or forensic purposes; because the design was retrospective and the images had already been acquired for clinical care, no additional clinical evaluation of the underlying indication was undertaken. Cases with knee pathology, prior surgical intervention, motion artifact, or images of otherwise insufficient diagnostic quality were excluded at the outset, so the analyzed sample reflects only diagnostic-quality examinations. After these exclusions, data were obtained for 309 individuals (153 males, 156 females) with an age range of 9.18–25.97 years (Table 1); mean age was 19.74 ± 3.84 years in males and 18.45 ± 3.88 years in females. The study protocol was approved by the hospital’s Ethics Committee of Health Sciences University, Bakırköy Dr. Sadi Konuk Training and Research Hospital, Department of Radiology (approval no. 04, approval date 8 May 2025).

Because the sample was drawn from clinically indicated examinations rather than a population-based cohort, and given the retrospective, single-center design, the number of participants in the youngest age strata (9–11 years; Table 1) is small—in some cells a single case or none at all—reflecting the age distribution of patients referred for knee MRI at this institution rather than a targeted sampling frame; findings in these strata are described in Section 4.5 (Limitations) as correspondingly imprecise. Whether sedation was required for any of the youngest participants was not systematically documented in the retrospective clinical records reviewed here and could not be verified; this is likewise noted as a limitation.

### 2.2. Data Acquisition

All examinations were performed using a 3-Tesla whole-body MRI system (Verio; Siemens Healthineers, Erlangen, Germany), with coronal three-dimensional Multiple Echo Data Image Combination (3D MEDIC) images (TR, 42 ms; TE, 18 ms; slice thickness, 1.5 mm; flip angle, 8°) used to evaluate the skeletal maturation of the distal femoral and proximal tibial epiphyses. MEDIC is a T2*-weighted spoiled gradient-echo sequence in which multiple gradient-recalled echoes, collected from a single excitation, are combined into a single composite image, improving signal-to-noise while reducing sensitivity to motion and flow artifact. In its three-dimensional implementation, the sequence is acquired as a single volumetric slab rather than sequential two-dimensional slice packages, yielding thin, contiguous, near-isotropic sections better suited to resolving the thin, obliquely oriented cartilage of the growth plate than conventional two-dimensional imaging.

**Figure 1:**
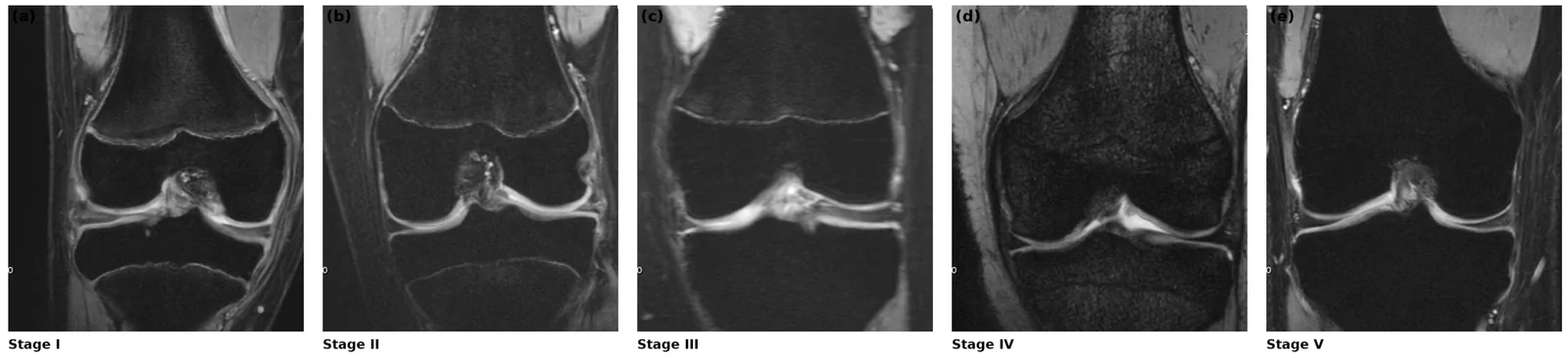
Coronal 3D MEDIC (Stage I–V) knee MR images illustrating the five Dedouit ossification stages of the distal femoral and proximal tibial epiphyseal cartilage. As an exception, the proximal tibial epiphysis in this same image has already reached Stage V (complete fusion), illustrating that the femur and tibia do not necessarily mature synchronously within the same individual.

Two observers—two experienced expert in skeletal radiology (R1) and an experienced radiologist (R2)—independently interpreted the images and assigned ossification stages using the original five-stage classification of Dedouit et al. [8] (Table 2; Figure 1), without the sub-stage modifications introduced in subsequent studies [13]. All images were re-evaluated after a four-week interval, blinded to the initial staging results, to permit assessment of intra- and inter-observer agreements.

### 2.3. Statistical Analysis

Intra- and inter-observer agreement was assessed using Cohen’s κ, with weighted κ values and agreement rates also calculated. Resulting κ values were interpreted according to the classification proposed by Altman [16]. Descriptive statistics (minimum, maximum, mean, standard deviation, lower and upper quartiles, and median age) were calculated for each stage, separately by sex and by anatomical site. The relationship between chronological age and ossification stage was assessed using Spearman’s rank correlation coefficient.

Between-sex differences in age at each stage were assessed using the Mann–Whitney U test; effect sizes were expressed as the rank-biserial correlation coefficient (r), and *p*-values were corrected for multiple comparisons across the eight stage-by-bone strata in which both sexes had at least two evaluable cases (Bonferroni correction, α = 0.05/8 = 0.00625). Comparisons that could not be computed because one sex had fewer than two cases (tibia Stages II and III) are reported descriptively without a statistical test.

To provide a threshold appropriate for forensic rather than purely descriptive use, we additionally fit a binary logistic regression of the probability of complete fusion (Stage V versus Stages I–IV) on chronological age, separately for each bone and sex, and solved for the age at which the model-predicted probability equaled 50% (age50); 95% confidence intervals for age50 were obtained by non-parametric bootstrap resampling (2000 resamples with replacement, stratified by bone and sex). We further computed the sensitivity, specificity, positive and negative predictive values, and the area under the receiver operating characteristic curve (AUC) for using Stage V as a binary predictor of chronological age ≥18 years, again separately by bone and sex.

A *p*-value < 0.05 (uncorrected) or below the Bonferroni-adjusted threshold, as specified, was considered statistically significant. Statistical analyses were performed in Python (version 3.11) using SciPy, statsmodels, and scikit-learn.

## 3. Results

### 3.1. Observer Agreement

Intraobserver agreement for staging of the distal femoral epiphysis was κ = 0.819, and interobserver agreement was κ = 0.808. For the proximal tibial epiphysis, intraobserver agreement was κ = 0.833 and interobserver agreement was κ = 0.821. According to the classification of Altman [16], all four values correspond to “very good” agreement, indicating that the original five-stage Dedouit classification was applied with high repeatability and consistency by both observers on the 3D MEDIC sequence.

### 3.2. Age and Ossification Stage

Descriptive statistics for each stage of the distal femoral and proximal tibial epiphysis, stratified by sex, are presented in Table 3 and Table 4, respectively, together with the between-sex Mann–Whitney comparison, its Bonferroni-corrected *p*-value, and the rank-biserial effect size (r) for each stratum with at least two cases per sex.

### 3.3. Sex Differences

After Bonferroni correction for the 8 testable stage-by-bone strata, only the between-sex age difference at Stage V remained statistically significant, for both the distal femoral (raw *p* < 0.001, corrected *p* = 0.004) and proximal tibial (raw *p* < 0.001, corrected *p* = 0.0008) epiphyses, with males consistently reaching Stage V at older ages than females (rank-biserial r = −0.274 and −0.294, respectively—a small-to-moderate effect). The raw difference at femoral Stage II (*p* = 0.015, r = −0.886, indicating a very large effect in a very small sample of five males and seven females) did not survive Bonferroni correction and should be regarded as exploratory rather than confirmatory, consistent with the sparse cell sizes noted in Section 4.5. No other stage comparison approached significance either before or after correction. Sample sizes at Stages II and III of the proximal tibial epiphysis were too small (N ≤ 1 per sex) to support any statistical comparison.

### 3.4. Age at 50% Probability of Complete Fusion (Stage V)

Because a minimum observed age is an extreme-value statistic that is highly sensitive to sample size and outliers, we additionally modeled the probability of complete fusion (Stage V) as a continuous function of chronological age and derived the age at which this probability reached 50%, with 95% bootstrap confidence intervals (Table 5). This age50 metric—rather than the minimum age reported in Section 3.6—is the recommended reference value for comparison with other cohorts and for probabilistic forensic reasoning.

### 3.5. Diagnostic Accuracy for the 18-Year Threshold

Table 6 presents the sensitivity, specificity, predictive values, and AUC of Stage V (versus Stages I–IV) as a binary indicator of chronological age ≥18 years. Sensitivity was uniformly high (0.96–0.98) in both sexes and bones, meaning that individuals younger than 18 years were rarely misclassified as Stage V (few false positives for adulthood arise from this direction). Specificity, however, differed markedly by sex: 0.77–0.85 in males but only 0.48–0.58 in females, indicating that a substantial proportion of females under 18 years had already reached Stage V and would be incorrectly classified as adult if Stage V were used as a sole criterion.

### 3.6. Minimum Age Thresholds

For the distal femoral epiphysis, the youngest age observed at Stage V (complete fusion) was 14.67 years in females and 16.16 years in males. For the proximal tibial epiphysis, the corresponding minimum ages at Stage V were 14.67 years in females and 16.07 years in males. Minimum age increased monotonically across stages for both epiphyses and both sexes (Table 3 and Table 4). As noted in Section 3.4, these single-observation minimum values are reported for comparability with prior studies that used the same metric, but they are statistically fragile—driven by a single individual and highly sensitive to sample size—and the probability-based age50 thresholds in Table 5 should be preferred wherever a defensible forensic estimate is required.

## 4. Discussion

### 4.1. Age Correlation and Observer Reliability

The present findings confirm a strong, monotonic relationship between chronological age and Dedouit stage for both the distal femoral and proximal tibial epiphysis (rho = 0.70–0.76), consistent in magnitude with correlations reported for other MRI-based knee staging protocols [13]. Intra- and inter-observer agreement (κ = 0.808–0.833) likewise fell within the “very good” range [16], confirming that the original five-stage classification—developed for spin-echo proton-density imaging [8]—remains reproducibly applicable when read directly from a 3D gradient-echo MEDIC sequence, without requiring the additional sub-stages that proved necessary for a two-dimensional gradient-echo sequence in our group’s earlier work [13].

### 4.2. Minimum Age Thresholds: Sequence or Population?

The central finding of this study concerns the age at which Stage V (complete epiphyseal fusion) becomes probable: the 50%-probability age (age50, Table 5) ranged from 15.23 years (tibia, females) to 17.43 years (femur, males), and even the extreme-value minimum ages (14.67 years in females, 16.07–16.16 years in males) were considerably younger than those reported in Dedouit et al.’s original description of the same five-stage system using FSE PD imaging, where Stage V was not observed before 22 years in either sex [8], and younger than those obtained with a two-dimensional multi-echo gradient-echo sequence applied to the same structures in our group’s earlier work [13].

Beyond the general T2*-versus-T2 distinction, the volumetric (3D) implementation of the MEDIC sequence used here introduces further technical factors that could plausibly bias the apparent timing of fusion specifically toward younger ages, rather than merely adding random noise around the true value. Spoiled gradient-echo sequences generate contrast without a refocusing pulse, leaving them intrinsically sensitive to intravoxel dephasing from local susceptibility gradients—an effect explicitly noted as a limitation of 3D gradient-echo cartilage protocols in general [17]. At the femoral and tibial growth plate, the interface between residual cartilage and adjacent trabecular bone constitutes exactly this kind of susceptibility boundary. As the physeal scar calcifies during the final stages of fusion, small trabecular spicules crossing the former growth plate could generate localized signal voids that “bloom” outward on a T2*-weighted acquisition, extinguishing the thin hyperintense cartilage line before it has, in histological terms, fully disappeared. If this mechanism operates, the Stage V criterion—no increased signal intensity between metaphysis and epiphysis—would be satisfied on MEDIC images at a genuinely younger biological stage than the same criterion applied to a spin-echo image, in which the 180° refocusing pulse actively cancels this static-field contribution.

Whether the volumetric acquisition specifically worsens or improves this effect relative to a 2D acquisition is less straightforward than it might first appear. On one hand, the thinner, contiguous sections obtained with 3D MEDIC (1.5 mm here, versus 3–3.5 mm for the 2D sequences in the same protocol) should reduce partial-volume averaging along the slice-select direction, which would be expected to preserve rather than obscure a thin residual cartilage line. On the other hand, 3D gradient-echo acquisitions are acquired as a single excited slab sampled with an additional phase-encoding dimension, over a correspondingly longer total acquisition time; comparative work in other joints has shown 3D gradient-echo sequences to be more susceptible to motion-related k-space corruption than 2D sequences, since motion during any part of a lengthy 3D acquisition can degrade the entire dataset rather than a single slice [18]. Separately, the very low flip angle typically used in 3D spoiled gradient-echo protocols to limit RF-induced saturation across a large excited volume—here, 8°, compared with much higher flip angles for the 2D sequences in the same protocol—alters the balance of T1, T2*, and proton-density weighting in a way that is not simply a scaled-down version of a 2D acquisition’s contrast, and could independently affect the conspicuity of a thin fluid-equivalent cartilage remnant against marrow fat. Chemical-shift misregistration at the fat–cartilage boundary, which affects gradient-echo sequences generally and depends on receiver bandwidth [19], could similarly shift the apparent margin of the growth plate, though this parameter was not systematically compared across sequences here. Taken together, these considerations suggest that a 3D multi-echo gradient-echo acquisition is not a straightforward, contrast-neutral upgrade of a 2D protocol for the specific purpose of growth-plate staging, even though it may offer advantages for general musculoskeletal image quality [17,18]; the possibility that volumetric T2*-weighted acquisition systematically advances the apparent age of fusion deserves direct testing.

A population-level explanation remains equally plausible. A prior Turkish sample assessed with FSE PD imaging reported comparably young thresholds for near-complete fusion (14–15 years in females, 15–16 years in males) [14], closer to the present findings than to Dedouit’s French [8] or Vieth et al.’s German [12] cohorts. Notably, the relevant literature does not attribute such differences to ethnicity or population identity per se—ethnicity has specifically been reported not to influence skeletal maturation once other factors are accounted for—but rather to socioeconomic status and associated nutritional and healthcare conditions, which are established determinants of the pace of ossification and have shifted over time even within the same population as living standards have changed [20,21]. Neither socioeconomic status nor comparable background variables were available in the clinical records reviewed here, so this explanation cannot be tested directly in the present sample, but it offers a more mechanistically grounded account of the discrepancy than population identity alone, and should be prioritized in any future prospective comparison.

A further source of variability worth acknowledging is the examiner. Staging systems of this kind are not free of observer-dependent variation, and prior work has shown that the qualification and experience of the examiner measurably affects the reliability of epiphyseal stage determination [22]. In the present study, both readers were experienced in musculoskeletal and forensic image interpretation, and the resulting intra- and inter-observer agreement was correspondingly high (Section 3.1); this makes examiner inexperience an unlikely explanation for the younger thresholds observed here, but it does mean that the present results describe the performance of the Dedouit system as applied by experienced raters and may not directly generalize to less experienced observers.

The present single-sequence, single-population design cannot fully adjudicate between a sequence-based and a socioeconomically mediated population effect; a within-subject comparison of imaging sequences, combined with prospectively collected socioeconomic data—and, where feasible, standardized metrics such as age50 rather than raw minimum ages for cross-study comparison—would be needed to separate these contributions.

### 4.3. Sex Differences in Fusion Timing

Females reached complete fusion (Stage V) at significantly younger ages than males for both the femur and tibia, a difference that survived Bonferroni correction and is consistent with the well-established pattern of earlier skeletal maturation in females. This pattern has a recognized endocrine basis: estrogen, rather than androgen, is now understood to be the principal driver of epiphyseal growth-plate senescence and fusion in both sexes, and the earlier attainment of pubertal estrogen levels in females relative to the later aromatization-dependent rise in males provides a physiological explanation for the sex gap observed here [23]. The single raw sex difference observed at an intermediate stage (femoral Stage II) did not survive correction for multiple comparisons and, given the very small subgroup sizes involved (five males, seven females), should not be interpreted as evidence of a genuine intermediate-stage sex effect; we report it only for transparency and completeness.

### 4.4. Forensic Application: The 18-Year Threshold

The diagnostic-accuracy results in Table 6 have a direct bearing on how these thresholds should, and should not, be used in practice. High sensitivity but sex-dependent, moderate-to-poor specificity mean that Stage V is a reasonably safe criterion for excluding adult status (a Stage I–IV finding is very unlikely in an adult) but a poor sole criterion for confirming it, particularly in females, where roughly two in five individuals under 18 years had already reached Stage V on this sequence. This asymmetry echoes a broader point made in the third-molar dental-age literature, where eruption-based criteria have likewise been found to carry a non-trivial risk of misclassifying minors as adults when a single anatomical site is used in isolation, and where the eruption stage of the third molar alone was similarly judged an imperfect standalone indicator for the 18-year threshold [7,24]; both bodies of evidence support combining multiple skeletal or dental sites, or reporting an explicit probabilistic threshold rather than a single categorical cut-off, when a forensic determination of majority is required. Distal femoral MRI findings have previously been proposed as a contributory, rather than standalone, criterion for the 18-year limit [24], a framing that the present sex-stratified specificity results directly support.

As in forensic dental age estimation, where the concept of an ethically and technically acceptable margin of error has been explicitly formalized for third-molar-based methods [6,7], we suggest that any adoption of the present MEDIC-based thresholds in medico-legal practice should be accompanied by the sex- and bone-specific error rates reported in Table 6, rather than by the age50 or minimum-age figures alone.

### 4.5. Limitations

First, the retrospective design produced an uneven distribution of cases across stages, with very few individuals captured at Stages II and III, particularly for the tibia (N = 1 in each sex); minimum age estimates at these stages should be interpreted with corresponding caution, and between-sex comparisons at these strata could not be statistically tested at all.

Second, only a single MRI sequence was evaluated, precluding a direct within-sample test of the sequence-versus-population question raised above.

Third, information on socioeconomic status and ethnicity was not available in the clinical records, preventing direct evaluation of what the literature identifies as the more likely driver of population-level differences in maturation timing.

Fourth, receiver bandwidth for the 3D MEDIC sequence was not recorded alongside the other acquisition parameters, precluding quantification of chemical-shift misregistration at the cartilage–marrow interface.

Fifth, although both observers were experienced, examiner-dependent variation is a recognized source of error in epiphyseal staging [22], and the present thresholds should be understood as specific to interpretation by experienced raters.

Sixth, all examinations were obtained for clinical indications (trauma, pain, or restricted range of motion) rather than as part of a population-based sampling frame; although cases with frank knee pathology, prior surgery, or motion artifact were excluded at the outset, we cannot fully exclude a residual influence of the underlying clinical indication on epiphyseal appearance, and the sample should not be assumed representative of the general Turkish adolescent and young-adult population.

Seventh, whether sedation was administered to any of the youngest participants was not systematically documented in the retrospective records and could not be verified; if used, sedation is not expected to affect cartilage signal but is noted here for completeness.

Eighth, information on physical activity or athletic participation—a factor reported elsewhere to be associated with skeletal maturation tempo—was not available in the clinical records and could not be examined as a covariate.

Finally, as with any single-center retrospective sample, generalization of these thresholds to the wider Turkish population should await replication in independent, ideally prospective, cohorts.

## 5. Conclusions

Applying the original, unmodified five-stage Dedouit classification to a volumetric 3D MEDIC gradient-echo sequence, this study found a strong correlation between chronological age and knee epiphyseal ossification stage, very good intra- and inter-observer reliability among experienced raters, and an age50 for complete fusion (Stage V) of 15.23–15.90 years in females and 16.96–17.43 years in males, with corresponding extreme-value minimum ages of 14.67 years in females and 16.07–16.16 years in males. Used as a binary predictor of the 18-year legal threshold, Stage V showed high sensitivity in both sexes but specificity that was markedly lower in females (0.48–0.58) than in males (0.77–0.85), meaning it should not be relied upon as a sole criterion for confirming adult status, particularly in female examinees. These thresholds were notably younger than those reported using spin-echo proton-density imaging in the original Dedouit cohort and using a two-dimensional gradient-echo sequence in prior work from our group, while aligning more closely with a Turkish sample previously assessed by spin-echo proton-density imaging. Both a sequence-specific technical explanation—rooted in the differing susceptibility to intravoxel dephasing, flip-angle behavior, and chemical-shift effects between spin-echo, 2D gradient-echo, and 3D gradient-echo acquisitions—and a socioeconomically mediated population effect offer plausible accounts of this discrepancy, and the present data cannot fully distinguish between them. Neither acquisition sequence nor population background should therefore be assumed negligible when applying existing MRI-based staging criteria to a new protocol or population without local validation, and any forensic use of these thresholds should incorporate the sex-specific error rates reported here rather than a single age cut-off.

## Figures and Tables

**Table 1:** Age distribution by sex.

Age (Years)	Male (N)	Female (N)
9	1	0
10	0	0
11	0	2
12	6	7
13	9	16
14	8	15
15	5	11
16	7	13
17	12	16
18	13	14
19	16	14
20	10	7
21	22	12
22	12	7
23	7	8
24	9	5
25	16	12

153 males, 156 females (N = 309).

**Table 2:** Definition of the original five-stage Dedouit MRI grading system.

Stage	Definition
I	A continuous horizontal cartilage layer thicker than 1.5 mm is present between the metaphysis and epiphysis, with a multilaminar appearance.
II	A continuous horizontal cartilage layer thicker than 1.5 mm is present, without a multilaminar appearance.
III	A continuous horizontal cartilage layer thinner than 1.5 mm is present.
IV	A discontinuous horizontal cartilage layer thinner than 1.5 mm is present.
V	The epiphyseal cartilage has fused completely and no epiphyseal scar remains visible.

Adapted from Dedouit et al. [8].

**Table 3:** Descriptive statistics for the distal femoral epiphysis, by stage and sex, with between-sex comparison.

Stage	Sex	N	Min	Max	Mean ± SD	Median	*p* (Raw)	*p* (Bonf.)	r
I	Male	28	9.18	16.45	13.84 ± 1.36	13.84			
I	Female	26	11.49	16.62	13.59 ± 1.24	13.46	0.253	1.000	−0.183
II	Male	5	15.09	17.14	16.18 ± 0.87	16.07			
II	Female	7	13.11	16.06	14.47 ± 0.95	14.31	0.015	0.118	−0.886
III	Male	3	15.44	17.19	16.60 ± 1.00	17.17			
III	Female	4	13.15	16.93	14.64 ± 1.84	14.25	0.108	0.867	−0.833
IV	Male	8	16.07	22.00	18.10 ± 1.82	17.77			
IV	Female	11	13.11	19.77	16.92 ± 1.93	16.81	0.215	1.000	−0.352
V	Male	109	16.16	25.97	21.62 ± 2.52	21.47			
V	Female	108	14.67	25.96	20.18 ± 3.22	19.74	<0.001	0.004	−0.274

LQ/UQ omitted for brevity from the previous version; SD not calculable for N = 1. *p* (raw): two-sided Mann–Whitney U, male vs. female age within stage. *p* (Bonf.): Bonferroni-corrected across the 8 testable strata (α = 0.00625). r: rank-biserial correlation (negative values indicate males reached that stage at older ages).

**Table 4:** Descriptive statistics for the proximal tibial epiphysis, by stage and sex, with between-sex comparison.

Stage	Sex	N	Min	Max	Mean ± SD	Median	*p* (Raw)	*p* (Bonf.)	r
I	Male	27	9.18	16.45	13.81 ± 1.37	13.83			
I	Female	24	11.49	16.62	13.53 ± 1.26	13.36	0.227	1.000	−0.199
II	Male	4	15.65	17.14	16.46 ± 0.71	16.52			
II	Female	1	13.72	13.72	13.72	13.72	not tested (n = 1)	—	—
III	Male	1	17.19	17.19	17.19	17.19			
III	Female	1	13.58	13.58	13.58	13.58	not tested (n = 1)	—	—
IV	Male	7	14.73	19.23	16.81 ± 1.85	16.87			
IV	Female	13	13.11	19.00	15.43 ± 2.15	14.89	0.132	1.000	−0.429
V	Male	114	16.07	25.97	21.46 ± 2.61	21.44			
V	Female	117	14.67	25.96	19.88 ± 3.29	19.34	<0.001	0.0008	−0.294

Stages II and III of the tibia include only 1 case per sex; between-sex testing is not statistically meaningful at this sample size and is reported descriptively only (see Section 4.5, Limitations). Spearman’s rank correlation showed a strong, statistically significant positive association between chronological age and ossification stage for both epiphyses. For the distal femoral epiphysis, correlation coefficients were rho = 0.739 overall (*p* < 0.001), rho = 0.763 in males (*p* < 0.001), and rho = 0.725 in females (*p* < 0.001). For the proximal tibial epiphysis, the corresponding values were rho = 0.702 overall (*p* < 0.001), rho = 0.740 in males (*p* < 0.001), and rho = 0.681 in females (*p* < 0.001).

**Table 5:** Age at 50% probability of complete fusion (Stage V), by bone and sex, with 95% bootstrap confidence intervals.

Bone	Sex	Age50 (Years)	95% CI
Femur	Male	17.43	16.95–17.95
Femur	Female	15.90	15.36–16.48
Tibia	Male	16.96	16.44–17.44
Tibia	Female	15.23	14.76–15.75

Age50: age at which a binary logistic model of *p* (Stage V | age) predicts a 50% probability of complete fusion. 95% CI from 2000 stratified bootstrap resamples.

**Table 6:** Diagnostic accuracy of Stage V for identifying individuals ≥ 18 years, by bone and sex.

Bone	Sex	N	Sens.	Spec.	PPV	NPV	AUC
Femur	Male	153	0.971	0.854	0.936	0.932	0.924
Femur	Female	156	0.962	0.584	0.704	0.938	0.782
Femur	All	309	0.967	0.688	0.820	0.935	0.837
Tibia	Male	153	0.981	0.771	0.904	0.949	0.882
Tibia	Female	156	0.975	0.481	0.658	0.949	0.732
Tibia	All	309	0.978	0.592	0.779	0.949	0.790

AUC calculated using the ordinal 5-level Dedouit stage as the predictor. PPV/NPV are sample-prevalence-dependent and should not be transferred to populations with a different age structure.

## Data Availability

The data presented in this study are available on request from the corresponding author, subject to institutional approval, due to ethical and privacy restrictions on patient imaging data.
